# Recognition of Fingerspelling Sequences in Polish Sign Language Using Point Clouds Obtained from Depth Images

**DOI:** 10.3390/s19051078

**Published:** 2019-03-03

**Authors:** Dawid Warchoł, Tomasz Kapuściński, Marian Wysocki

**Affiliations:** Department of Computer and Control Engineering, Faculty of Electrical and Computer Engineering, Rzeszów University of Technology, W. Pola 2, 35-959 Rzeszów, Poland; dawwar@kia.prz.edu.pl (D.W.); mwysocki@kia.prz.edu.pl (M.W.)

**Keywords:** hand posture recognition, fingerspelling, Polish finger alphabet, American finger alphabet, Kinect, point cloud, hidden Markov models

## Abstract

The paper presents a method for recognizing sequences of static letters of the Polish finger alphabet using the point cloud descriptors: viewpoint feature histogram, eigenvalues-based descriptors, ensemble of shape functions, and global radius-based surface descriptor. Each sequence is understood as quick highly coarticulated motions, and the classification is performed by networks of hidden Markov models trained by transitions between postures corresponding to particular letters. Three kinds of the left-to-right Markov models of the transitions, two networks of the transition models—independent and dependent on a dictionary—as well as various combinations of point cloud descriptors are examined on a publicly available dataset of 4200 executions (registered as depth map sequences) prepared by the authors. The hand shape representation proposed in our method can also be applied for recognition of hand postures in single frames. We confirmed this using a known, challenging American finger alphabet dataset with about 60,000 depth images.

## 1. Introduction

Recognition of static hand posture sequences is a natural and relevant expansion of static hand posture recognition. Changing one hand shape to another, e.g., by performing two finger alphabet letters represented by different static hand postures, introduces motion of the hand that is not the part of a gesture itself. Recognition of static hand posture sequences is often compared to recognition of speech, which consists of phoneme sequences. However, in the case of hand gestures, the recognition problem is more difficult because there are no silence models, which are present in audio signals.

Recognition of static hand postures in single images, point clouds or skeletons is a common problem. Researchers have addressed this issue using different algorithms [1,2,3,4,5,6,7]. However, recognition of static hand posture sequences is an issue rarely addressed in the literature. A recognition algorithm can work based on two strategies: (i) Skipping motion related to transitions between postures and (ii) exploiting motion information. The first of these strategies was used by Lamar et al. [8] and Oz et al. [9], where recognition of American and Japanese sign language alphabets was achieved by discarding frames with motion and choosing only frames corresponding to letters. Skipping motion frames is performed by calculating the difference between consecutive frames [8] or direct measure of hand velocity using sensory gloves [9]. This approach is problematic in the case of gestures fingerspelled at high speed (which is natural for proficient signers) because the pauses, during which the hand shape is fixed in consecutive frames, are very short or do not exist at all. In many works [10,11,12,13,14,15], hidden Markov models are used for recognition of letter sequences. There are models based on letters [10,12,13], based on transitions between consecutive letters [14,15], and based on phonological features of fingerspelling [11,12]. Some of the mentioned approaches require a signer to wear a special outfit—long sleeves [10,13] or gloves [8,9]. Liwicki et al. [13] recognized letter sequences of the British finger alphabet that were performed with both hands. Such gestures provide information which is more useful in distinguishing between classes than in the case of single-handed gestures. The works mentioned above use different kinds of data: Grey images [14], color images [8,10,11,12,13], hand skeletons acquired by a Leap Motion camera [15], and finger joint angles obtained from sensory gloves [9]. The following finger alphabets are recognized: American [10,11,12,14], Japanese [8], British [13], and German [15]. All of the mentioned works involve data other than point clouds or depth images, and none of the works are related to Polish finger alphabet.

In this work, we present an approach to recognition of Polish finger alphabet letter sequences using 3D data in the form of point clouds. Three-dimensional data have several advantages over data formats used in the mentioned works. One of the advantages is the possibility of hand segmentation based on depth that can be more accurate than methods relying solely on color information. Another advantage is the representation of objects using features based on the surface geometry in addition to the standard 2D shape features. Moreover, 3D cameras using time-of-flight technology work correctly under poor lighting conditions or even in absolute dark. Additionally, users performing gestures are not required to wear any gloves or to keep their hands very close to the camera, as is the case with Leap Motion cameras. In addition to the listed advantages, the main motivation for using 3D data is the good results obtained for the recognition of static hand postures in single images with representative datasets [16,17,18].

**Figure 1:**
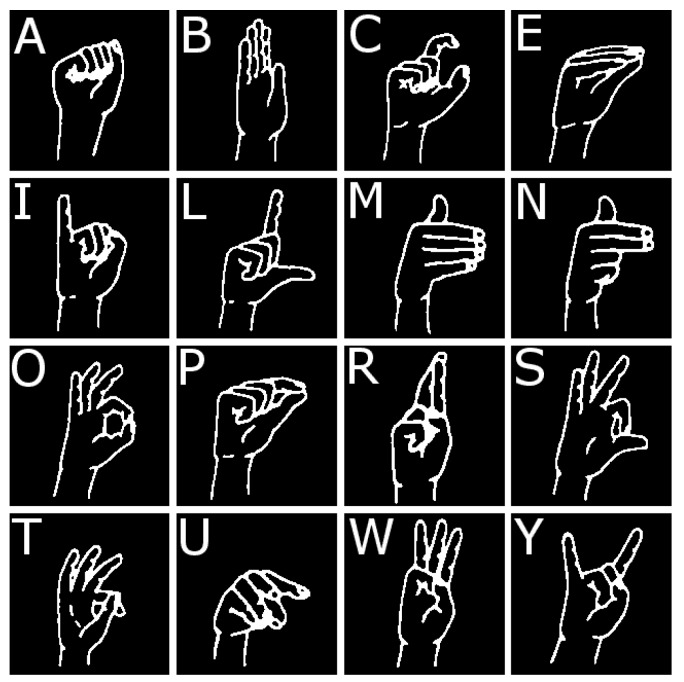
Letters of Polish finger alphabet represented by static postures.

For gesture classification, we used hidden Markov models and the strategy exploiting motion related to transitions between letters. Our feature set was calculated using the point cloud descriptors: viewpoint feature histogram (VFH) [19], eigenvalues-based descriptors [20], and modified versions of two other descriptors: Ensemble of shape functions (ESF) [18,21] and global radius-based surface descriptor (GRSD) [18,22]. We focused on every letter from the Polish finger alphabet that is represented by a static hand posture: A, B, C, E, I, L, M, N, O, P, R, S, T, U, W, Y. Hand postures representing this set of letters are shown in Figure 1. It is worth noting that some of the postures are difficult to distinguish even for a human, e.g., letters O, S, T, or E, P. That makes recognition of Polish finger alphabet letters a challenging task.

The novelty of the paper is the study of recognition of finger alphabet letter sequences based on models of transitions between the letters built using point clouds and point cloud descriptors. The main contributions are:

1. Proposition of combining VFH, ESF, GRSD, and eigenvalues-based point cloud descriptors to build hand shape representations;
2. Experimental selection of the best descriptor combination and the best structure of hidden Markov models representing the hand shape sequences forming transitions between the pairs of letters;
3. Experimental verification of two recognition methods: Dependent and independent from the dictionary of continuous fingerspelled expressions (acronyms);
4. Experimental verification of the suitability of the proposed hand shape representation to recognition of hand postures in single frames by using a known, challenging American finger alphabet dataset with about 60,000 depth images;
5. Providing the dataset and the details of the proposed method (at this time, we do not know any other publicly available dataset containing depth maps (point clouds) of continuous fingerspelled expressions including a set of transitions between the pairs of letters).

The remaining part of the paper is organized as follows. Section 2 discusses the proposed recognition method. In Section 3, we describe the prepared datasets and our experiments with their results. Using the challenging American finger alphabet dataset [23], we show that the hand shape representation proposed in our method can also be applied for recognition of hand postures in single frames. Conclusions and proposals for future work are included in Section 4.

## 2. Recognition Method

This section presents the outline and the details of the proposed method.

### 2.1. Outline of the Method

**Figure 2:**
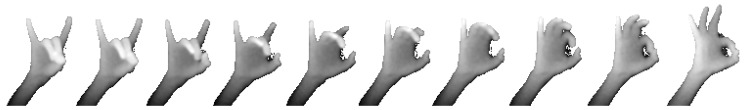
Several consecutive depth maps showing an exemplary transition from the letter Y to the letter O.

In this paper, we understand fingerspelling as quick highly coarticulated motions, not as the careful articulations of isolated signs. For recognition, we used the strategy exploiting motion related to transitions between the letters. The transition is called the sequence of hand shapes that appear between two consecutive letters (see Figure 2).

We modeled such sequences by hidden Markov models; thus, each pair of consecutive letters considered in this study is represented by such a model, prepared using the training data. Multi-letter expressions (e.g., acronyms) were modeled as networks of the transition models.

The proposed recognition algorithm consists of the following main steps:

1. Hand segmentation (based on depth map);
2. Building the point cloud of the hand, downsampling of the cloud, determining its bounding box, and decomposing the bounding box into smaller cells;
3. Extraction of features based on point cloud descriptors;
4. Recognition using hidden Markov models (after performing steps 1–3 for each frame of the video related to an expression being recognized).

Steps 1–3 were implemented using the OpenCV 2.4.9 [24] and the PCL 1.8.0 [25] programming libraries. For the classification, we used HTK 3.4.1 (hidden Markov model toolkit [26]), a software for building and manipulating hidden Markov models.

### 2.2. Hand Segmentation

The hand segmentation method was proposed in our previous work [17]. It begins with the extraction of the arm region (hand and forearm) from the depth map DM, which is our input data. For this purpose, the pixel with the lowest depth value (closest to the camera) is localized and marked as na and its depth, in meters, is denoted as $d_{na}$. Then, all the pixels with a depth value greater than $d_{na}+T_{depth}$ are rejected (set to 0), resulting in a depth map DA representing the arm. In our experiments, we set $T_{depth}$ to 0.1 m according to the empirical observations. Then, we threshold DA to obtain a binary image BA:

(1) $$BA_{i}=\left\{\begin{array}{cc} 1 & \mathrm{if}\;DA_{i}^{z}>0\\ 0 & \mathrm{if}\;DA_{i}^{z}=0 \end{array}\right.,$$

where $DA_{i}^{z}$ is the depth value of the i-th DA pixel and $BA_{i}$ is the *i*-th BA pixel.

To remove defects resulting from measurement errors of a camera, morphological operations should be performed on BA before the next segmentation stages. First, the jagged edges are smoothed using opening and closing with a 3×3 structuring element. For this operation, the functions *erode* and *dilate* from OpenCV library can be used. Then, small gaps within the arm region are filled if they are not larger than the rectangular structuring element of size 35×35 pixels. This size is sufficient for the data acquired by a Kinect camera in good lighting conditions or a Kinect 2.0 camera.

The next step of the hand segmentation is the selection of the central hand point. To this end, we applied the Gaussian filter on BA using the kernel of a large size Gk that can be calculated as follows: $Gk_{x}=Gk_{y}=round_{o}\left(100/d_{na}\right)$ pixels, where $round_{o}$ is a function rounding the value in parentheses to the nearest odd integer. For the Gaussian filter, OpenCV function *GaussianBlur* can be used. With such calculated Gk, the kernel covers the entire hand region. The resulting grayscale image has a global maximum (the brightest pixel) $C_{o}$ at the object’s center. To ensure that $C_{o}$ belongs to the hand (not to the wrist or the forearm region), we created a set *M* containing every local maximum not smaller than 85% of the $C_{o}$ value. Let us denote by $D_{\left(m,na\right)}$ the Euclidean 2D distance (on the xy plane) from the maximum point *m* to na and by $m_{v}$ the value of *m*. As a central hand point $C_{h}$, we selected a maximum from *M*, for which the difference $D_{\left(m,na\right)}-m_{v}$ is minimal. Thus, the hand center is not too far from the point of the lowest depth na, and it has a similar value to the global maximum $C_{o}$.

After the calculation of the hand center, the palm region of the hand is roughly estimated by the largest circle that can be fitted on it. Let us denote the circle with the center $C_{h}$ as $C_{c}$, and its radius as $RC_{c}$. The circle $C_{c}$ is iteratively enlarged, starting with $RC_{c}=1$ until the hand area inside it, denoted as $HC_{area}$, is less than 95% of the circle area $C_{area}$. Then, the circle is moved to a neighboring position maximizing the $HC_{area}$, and finally, the radius is increased once more. After this step, we obtain the updated central hand point $C_{h}^{\prime }$ with coordinates $C_{hx}^{\prime },\;C_{hy}^{\prime }$.

The last segmentation step is the rejection of the pixels corresponding to the forearm. For this purpose, each non-zero pixel of BA, whose *y* coordinate is less than $C_{hy}^{\prime }-RC_{c}$, is set to 0.

This method was designed for recognition of static postures in single point clouds (or depth images). For some hand postures, the circle can be fitted in a wrist region instead of palm, which leads to incorrect segmentation. Therefore, the algorithm is slightly modified to increase its effectiveness in posture sequence recognition. The modification includes checking whether the distance between the circle center $C_{c}$ and the highest hand point is more than 2.5 times (value determined experimentally) greater than the circle radius $C_{r}$. If this condition is satisfied, then the circle is considered as incorrectly fitted and its center is set to $C_{c}$ from the previous frame of the sequence (provided that the current frame is not the first).

### 2.3. Conversion from Depth Map to Point Cloud

After the hand segmentation, depth maps have to be converted into the point cloud format. To this aim, we defined the set *H* of hand pixels belonging to binary image BH:H={x∈BH|x=1}. The conversion was applied to each DA pixel if *H* contains a pixel of the same coordinates. Therefore, the resulting point cloud consists only of points belonging to the hand, without forearm and isolated objects. The coordinates of cloud points: $PCh_{i}^{x}$, $PCh_{i}^{y}$, and $PCh_{i}^{z}$ are set with respect to the DA pixels’ depth value $DA_{i}^{z}$ based on the perspective projection equations and the camera’s intrinsic parameters:

(2) $$PCh_{i}^{x}=\frac{\left(DA_{i}^{z}+fl\right)\cdot \left(\frac{DA_{width}}{2}-DA_{i}^{x}-1\right)\cdot ps_{x}}{fl},$$

(3) $$PCh_{i}^{y}=\frac{\left(DA_{i}^{z}+fl\right)\cdot \left(\frac{DA_{height}}{2}-DA_{i}^{y}-1\right)\cdot ps_{y}}{fl},$$

(4) $$PCh_{i}^{z}=DA_{i}^{z},$$

where $DA_{width}$ and $DA_{height}$ are the number of depth map columns and rows, respectively; fl is the focal length of a camera; $ps_{x}$ and $ps_{y}$ are the pixel’s width and height, respectively. The intrinsic parameters of the Kinect 2.0 camera, used in our experiments, are as follows: fl = 3.657 mm, $ps_{x}$ = $ps_{y}$ = 0.01 mm. These values were obtained using the camera calibration toolbox for Matlab software [27] (Caltech, Pasadena, CA, USA).

The resulting point cloud is redundantly dense. There is no need for such a large number of points to calculate representative features. Therefore, the cloud has to be downsampled in order to reduce the number of points and to speed up the process of feature calculation. This operation is performed by creating a 3D voxel grid over the input point cloud data. Each point situated within each voxel (3D cuboid) is approximated by its centroid. For this purpose, the PCL class *downsampler* can be used. The voxel dimensions (width, height, depth) $V_{x}\times V_{y}\times V_{z}$ are the filter parameters. We used cubic voxels and set the dimensions as follows: $V_{x}=V_{y}=V_{z}=4.5$ mm, which in our previous work [18] turned out to be the optimal value in terms of gesture recognition rate and feature extraction time.

### 2.4. Bounding Box

**Figure 3:**
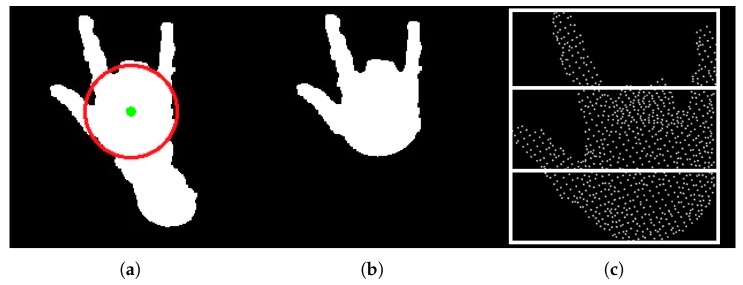
(**a**) Binary image of an arm with the circle *C* fitted in the palm region and circle center $C_{c}$ marked as a green dot; (**b**) binary image of a hand after rejecting the wrist region; (**c**) point cloud of a hand and its bounding box divided into three horizontal cells (projection on the *x*, *y* plane).

In our method, the bounding box, defined as the smallest possible axis aligned cuboid that entirely embraces the point cloud of the segmented hand, is determined before feature calculation. To increase the distinctiveness of calculated hand features, we divided the bounding boxes into several cuboidal cells of equal sizes. Our feature vectors consist of features calculated for each cell. The experiments performed in our previous works [16,17] showed that dividing the bounding box into three horizontal cells was most effective in the hand posture recognition (three vertical, and nine cells have also been tested). Therefore, in this work, we divided the box into three horizontal cells (see Figure 3c).

### 2.5. Point Cloud Descriptors

To calculate hand features, we used the following descriptors: viewpoint feature histogram, ensemble of shape functions, global radius-based surface descriptor, and eigenvalues-based descriptors.

The viewpoint feature histogram is a global descriptor of a point cloud considered as a set of 3D points in a clockwise coordinate system with the horizontal *x* axis directed to the left, the vertical *y* axis facing up, and the *z* axis coinciding with the optical axis of the camera and turned towards the observed objects. VFH consists of a surface shape component and a viewpoint direction component which is not used in our work, since it does not provide information useful in posture classification. The surface shape component has a form of four histograms describing distributions of the four parameters *d*, θ, α, and ϕ characterizing each point of the cloud. *d* means the distance between a point and the cloud centroid. The remaining parameters are angles computed based on an estimation of the vector normal to the surface at a given point and the average of such vectors for all points of the cloud hooked at the centroid [19].

The ensemble of shape functions is a combination of three functions describing the following properties of a point cloud: Distances, areas, and angles formed by connecting three randomly selected points. These values are placed in three histograms: IN, OUT, and MIXED, depending on whether the line connecting the points lies entirely inside the surface, entirely outside it, or both [21].

The global radius-based surface descriptor calculates the radial relationships between the neighboring points. For each pair of point and its neighbor, the descriptor calculates the distance between them and the difference between surface normals computed at these points. Based on this information, a sphere is defined, which is then used for the surface categorization. This process assigns one of several categories to each point and creates histograms based on the transitions between the categories [22,28].

We used modified versions of ESF and GRSD descriptors. The modifications were proposed in our previous work [18]. The main ESF changes include replacing IN and OUT histograms with MOSTLY-IN and MOSTLY-OUT histograms, which better differentiate between subtle changes in hand posture geometry. In modified GRSD, we proposed to combine the surface categories and to calculate features based on the combinations of categories instead of transition histograms. The modified descriptors are called ESF2 and GRSD-C. VFH, ESF, and GRSD with their modifications are described in detail in our previous works [17,18].

Eigenvalues-based descriptors are computed as follows. The 3D covariance matrix is constructed for a given 3D point cloud. Since the matrix is symmetric and positive–semidefinite, its three eigenvalues ($\lambda_{1},\lambda_{2},\lambda_{3}\in R,\lambda_{1}\geq \lambda_{2}\geq \lambda_{3}\geq 0$) exist, are real-valued, non-negative, and correspond to an orthogonal system of eigenvectors. The three eigenvalues represent the extent of a 3D ellipsoid along its principal axes. The normalized eigenvalues $e_{i}=\lambda_{i}/\left(\lambda_{1}+\lambda_{2}+\lambda_{3}\right)$; $e_{i}\in \left[0,1\right]$; i∈1,2,3; $e_{1}+e_{2}+e_{3}=1$ are used to define seven measures [20]: Linearity *L*, planarity *P*, scattering *S*, omnivariance *O*, anisotropy *A*, change of curvature *C*, and eigenentropy *E*.

Linearity *L*, planarity *P*, and scattering *S* represent the dimensionality features as:

(5) $$L=\frac{e_{1}-e_{2}}{e_{1}},$$

(6) $$P=\frac{e_{2}-e_{3}}{e_{1}},$$

(7) $$S=\frac{e_{3}}{e_{1}}.$$

Omnivariance *O* and anisotropy *A* can be computed as:

(8) $$O=\sqrt[{3}]{e_{1}e_{2}e_{3}},$$

(9) $$A=\frac{e_{1}-e_{3}}{e_{1}}.$$

Change of curvature *C* estimates the surface variation as:

(10) $$C=\frac{e_{3}}{e_{1}+e_{2}+e_{3}}=e_{3}.$$

Eigenentropy *E* may be defined by the Shannon entropy as:

(11) $$E=-e_{1}ln\left(e_{1}\right)-e_{2}ln\left(e_{2}\right)-e_{3}ln\left(e_{3}\right),$$

where the occurrence of eigenvalues identical to zero has to be avoided by adding an infinitesimally small value ε. *E* represents a measure describing the order (disorder) of 3D points of the considered point cloud. For justification of Equation (11), it is worth noting that the normalized eigenvalues satisfy two of three probability axioms according to Kolmogorov, and they may be considered as the probabilities, taking into account that quasi-probability distributions generally relax the third axiom addressing the junction of mutually disjoint random events [20].

We proposed to represent the calculated histograms of each histogram-based descriptor (VFH and ESF2) by their means and standard deviations. The features of GRSD-C and eigenvalues-based descriptors are not histograms but single values, and therefore, there is no need to calculate their mean and standard deviation. The results of our previous work on recognition of static hand gestures [17,18] justify using only two VFH histograms, denoted in the original work as ϕ, *d* (related to angles between the average of the surface normals hooked at the centroid of the point cloud and lines connecting the centroid with surface points, and distances between the centroid and surface points, respectively), one ESF2 histogram, denoted in original work as A3 (related to angles formed by lines connecting points) and two combined categories of GRSD-C: (i) Plane, cylinder, rim, sphere, and (ii) sharp edge, noise. These features are the most effective in terms of hand posture recognition rate in single images, which has been experimentally verified in our previous works. The selection of eigenvalues-based descriptors is described in Section 3.2. All features are normalized to the interval [0-1] using the extreme values estimated from the training set. The source codes for ESF2, GRSD-C, and eigenvalues-based descriptors can be downloaded from http://vision.kia.prz.edu.pl. The implementation of VFH descriptor is available in the PCL library.

### 2.6. Hidden Markov Models

The classification of posture sequences is carried out using hidden Markov models (HMMs). HMMs are useful for modeling the temporal structure and statistical properties of signals. They also find wide applications in gesture recognition (see, e.g., [29]). A hidden Markov model consists of two stochastic processes: (i) Unobservable Markov chain with a finite number of states, distribution of initial state probabilities, and a matrix of state transition probabilities; (ii) a set of probability density functions that are related to observations generated by states. Training of the models consists of parameter estimation based on observation sequences. For this purpose, we used the dynamic-programming Viterbi algorithm [30] implemented in the HTK software, with default parameters. This method tries to find a stochastic process (model) which generates observation sequences from the training set. In the classification stage, the algorithm returns a class represented by a model with the highest probability of generating the sequence to be recognized.

**Figure 4:**
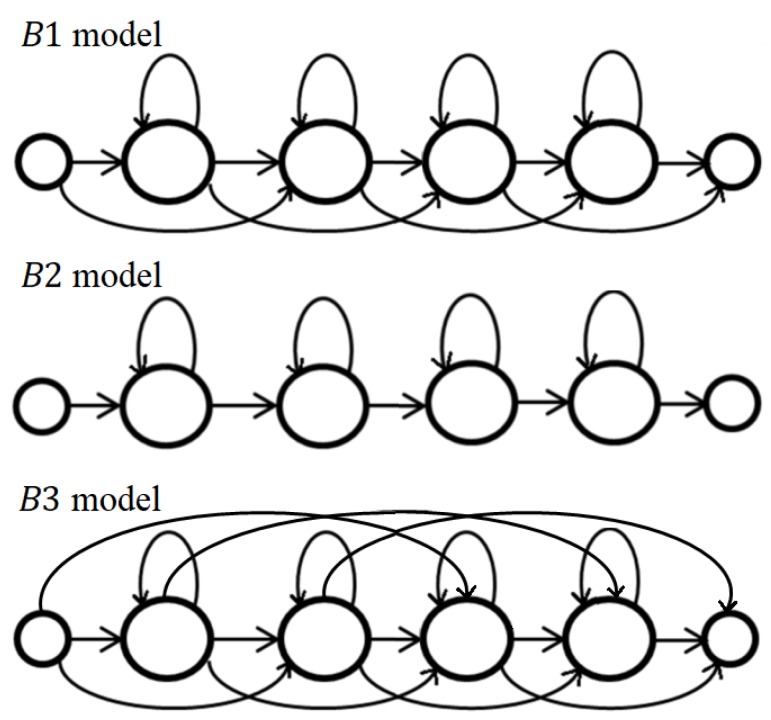
The state transitions diagrams of exemplary four-state models B1, B2, and B3. Smaller circles represent non-emitting states.

In the proposed recognition method, we used HMMs that represent transitions between particular alphabet letters (two-element combination of letters without repetition). Such an approach allows to exploit not only shape information of the hand showing particular letters, but also the motion related to transitions between letters. For this purpose, we considered three left-to-right models, also known as Bakis models (see Figure 4):

- B1—in which transitions to the respective next state and to the current state itself, as well as the skipping of individual states within a sequence, are possible. The self-transitions make it possible to capture variations in the temporal extension of the patterns described. Skipping of individual states allows the omission of short portions of a signal in its statistical description.
- B2—without the possibility of skipping individual states (also known as linear model).
- B3—like B1 with additional connections omitting two consecutive states. Thus, longer parts of the data to be processed may be missing.

The number of states, also called emitting states, because they generate observations, was set experimentally (see Section 3). For every emitting state, we used the Gaussian distribution with a diagonal covariance matrix as a probability density function. Additionally, the models contained two non-emitting states—the first and the last state—which were helpful in building model networks and required in the HTK software.

## 3. Experiments

In this Section, we describe the prepared dataset and list the recognized acronyms. Then, we describe the performed experiments and present their results.

### 3.1. Dataset

We verified our method on a dataset recorded by three people (named as person I, person II, and person III). The gestures were recorded using a Kinect 2.0 camera with Kinect for Windows SDK 2.0 [31]. It is a time-of-flight 3D camera with a USB 3.0 interface, which records depth maps with a resolution of 512×424 pixels at 30 frames per second. The camera was placed on a tripod at a distance of about 90 cm from a sitting signer and at a height of about 90 cm (Polish sign language letters are presented on the right side of the head). The speed of fingerspelling varied depending on the person. For person I, it was about 140–150 letters per minute, for person II, about 160–170 letters per minute, and for person III, about 90–100 letters per minute. Person I and person III learned gestures for the needs of dataset recording, whereas person II is a professional sign language translator. Each person performed gestures corresponding to transitions between letters five times, and thus, the training set consists of 3600 sequences. The testing set consists of the following twenty sequences of letters: ABW, ASL, CBOS, CEA, EUR, MOPS, MSW, NATO, NBP, NCBIR, NCN, PAN, PAP, PIS, OBOP, PLN, PO, PRL, RP, and WYO. Each acronym was performed ten times by each person, which gave 600 sequences in total. The content of the dataset is described in Table 1. The dataset can be downloaded from http://vision.kia.prz.edu.pl.

### 3.2. Classification without Knowledge of the Dictionary

**Figure 5:**
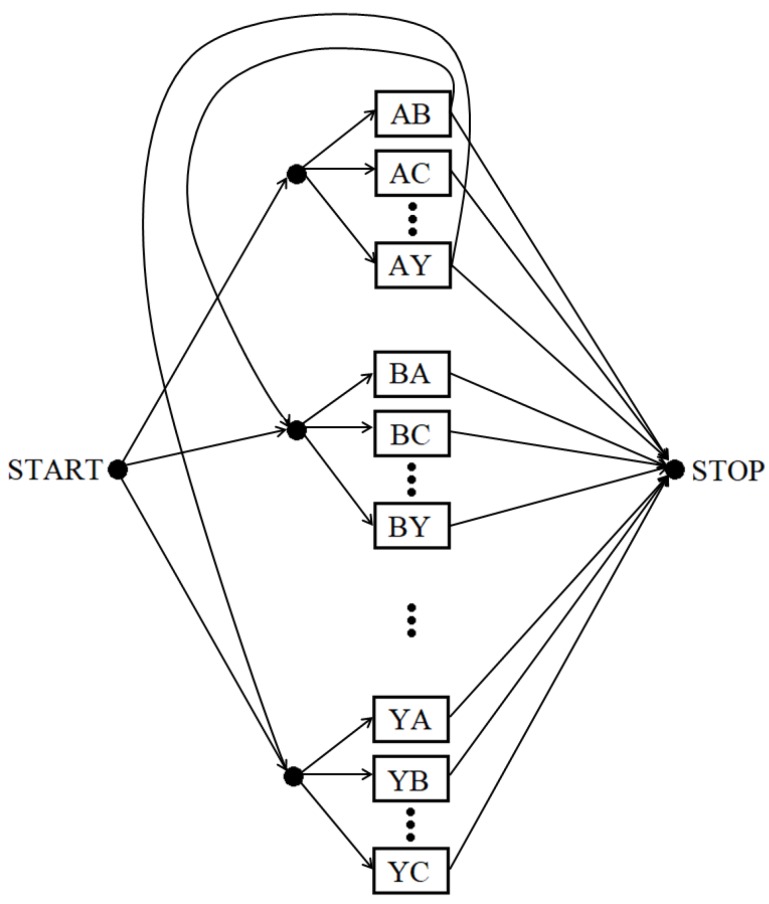
The structure of connected transition models for the method independent from the acronym dictionary. Every transition model is represented by a rectangular block. For the sake of clarity, only a few of all the blocks and connections are depicted.

In the first stage of experiments, the posture sequences were classified without knowledge of the considered words (the acronym dictionary was not used). For this purpose, we used a structure of connected transition models in which the first letter from each transition is equal to the second letter from the previous transition (see Figure 5). For example, if the recognized transition is AB, then the next transition must start with the letter B.

At this stage, each testing sequence was classified using models trained by all the sequences from the training set (i.e., the letter transitions). Because of the used HMM structure, in which words are created from the letter transitions, each letter, with the exception of the first and last one, was recognized twice. Therefore, each letter with even index, with the exception of the last letter, had to be deleted from the resulting sequences. For example, the sequence of the correctly recognized acronym NATO was initially identified as NAATTO. Deletion of the second and fourth letter yields the original sequence: NATO.

The recognition rate of the algorithm independent from the acronym dictionary was calculated using a method described by Kim et al. [11]—the rate of correctly recognized letters. The method consists of calculating the Levenshtein distance [32] of the recognized word from the original word corresponding to the given data sequence. The Levenshtein distance expresses the total number of single-character insertions, deletions, and substitutions. The calculated distance is then divided by the length of the original word yielding the letter error rate Er of the sequence. The average error rate ErM is calculated for the whole testing set and, finally, the rate of correctly recognized letters is calculated as: RM=1−ErM.

We chose this form of letter recognition rate because, in the case of a standard word recognition rate, the results seem to be significantly underestimated if only the single letters in the whole sequence are incorrectly recognized, inserted or omitted. In such cases, the standard word recognition rate yields the 100% error rate. The rate of correctly recognized letters results in a smaller error, depending on the length of the word. It is worth noting that when a recognized word is quite different (in terms of Levenshtein distance) than the original word, the rate of correctly recognized letters yields the error greater than 100%. Therefore, in such cases, the results can be less favorable than those of the standard word recognition rate.

We observed that the optimal number of states depended on the model type. Therefore, we performed recognition tests separately for models B1, B2, and B3 with a varying number of emitting states, ranging from 2 to 13. This experiment allowed us to set the number of states for each model: B1—11 states, B2—7 states, and B3—9 states.

We decided to choose the optimal set of eigenvalues-based descriptors since we did not use them in our previous works with posture recognition in single images. To this aim, we performed tests with the feature set consisting of all the eigenvalues-based descriptors (further marked by FS1) and the feature set consisting of all the remaining descriptors mentioned in Section 2.5 (VFH, ESF2, GRSD-C) – further marked by FS2. The average recognition rates for FS2 were 17.7% higher than for FS1. Then, we performed seven tests with feature sets containing single eigenvalues-based descriptors (one descriptor per test) and sorted them in descending order by recognition rate (see Table 2). We combined the set FS2 with the best eigenvalues-based descriptor, *E* (eigenentropy), and observed improvement of the results up to 5.5%, depending on the person from the testing set. Then, we tried adding the other best eigenvalues-based descriptor and observed that *O*, despite being the second best, does not work well when combined with other descriptors. Finally, we chose the most effective subset of FS1: The first (*E*), the third (*C*), the fourth (*L*), and the fifth (*S*) best eigenvalues-based descriptor. The results of the aforementioned experiments are presented in Table 3. Since GRSD-C was the least effective of the three descriptors analyzed in our previous works, we tried removing GRSD-C features from FS2; however, the results were not better. The final feature vector, used in the subsequent experiments, consists of the following components: VFH(ϕ,*d*), ESF2(A3), GRSD-C, and eigenvalues-based descriptors (*E*,*C*,*L*,*S*). Thus, the complete feature vector representing a video frame contains 16×3 features: Mean and standard deviation related to each of the two mentioned histogram-based descriptors, two values of GRSD-C descriptor, four eigenvalues-based descriptors, for each cell of the bounding box of the hand (see Figure 3c).

In Table 4, we present recognition rates of the method independent from the acronym dictionary for models B1, B2, and B3. The best mean recognition rates were obtained for B2; however, the standard deviation of the results turned out to be relatively high. The interesting fact is that B2 yields significantly higher recognition rate for person I and III, whereas B1 gives higher rates for person II (proficient signer).

The most misclassifications were related to visually similar letters, such as S mistaken for O and N mistaken for P. These mistakes are common in the case of posture recognition in single images. Other frequent mistakes were related to letters N and A as well as inserting additional, single letters at various positions of a sequence despite recognizing other letters correctly (e.g., MOSPS instead of MOPS).

### 3.3. Classification Using a Dictionary of Acronyms

**Figure 6:**
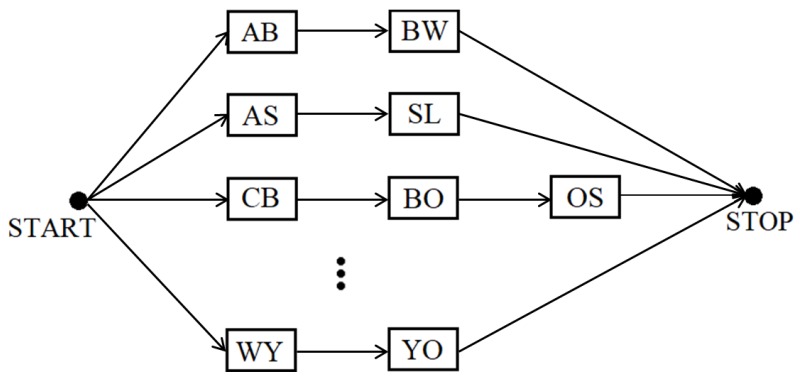
The structure of connected transition models for the method dependent on the acronym dictionary. Every transition model is represented by a rectangular block. For the sake of clarity, only a few of all the blocks and connections are depicted.

In the second stage of experiments, the posture sequences were classified by the method using a dictionary of acronyms from the testing set. Each testing sequence was classified using models trained by all the sequences from the training set. For this purpose, we used a structure of connected models containing only the connections corresponding to the classified acronyms (see Figure 6). The classification result is chosen from the twenty considered acronyms.

Recognition rates of this method are presented in Table 5. Experiments with a varying number of emitting states allowed us to set the following values for the recognition method using the acronym dictionary: B1—6 states, B2—8 states, and B3—3 states. It is worth noting that the average optimal number of states for the method using a dictionary of acronyms is smaller than for the method independent from the dictionary. The best mean recognition rates were obtained for the models B1 and B3 (only 0.1% difference); however, B1 yielded lower standard deviation. Similarly to the previous experiment, the worst results were obtained for person II.

In the third stage of experiments, we performed leave-one-person-out tests (see Table 6) for the method using the dictionary of acronyms: In each test, the person from the testing set was not present in the training set. For models B1 and B2, the recognition rates were almost equal and significantly higher than for model B3. The results of this stage can be considered satisfactory because of the difficulty of the performed tests. Standard deviations are larger than in the previous experiment stage, which results from the relatively low recognition rate obtained for person II.

The most frequent misclassification was incorrect recognition of acronym CEA in the case of person I and II. Most often, it was mistaken for PAP, PLN (person I), and RP (person II). The reason for this error could be a characteristic motion of thumb and index finger towards each other that can be observed in each of these acronyms. This motion is more visible in gestures performed by person I and II than by person III. Moreover, acronym NCN shown by person II was incorrectly recognized multiple times as NBP and RP. This can be partially explained by the difficulty in distinguishing between the similar letters N and P previously observed in the method independent from the acronym dictionary.

The difference between mean recognition rates of tests presented in Table 5 and leave-one- person-out tests presented in Table 6 is about 8%. It can be presumed that extending the training set with data recorded by more people would increase the classifier generalization ability, and thus improve the recognition rates of leave-one-person-out tests.

Finally, we performed an additional experiment with the testing set recorded by person IV, who showed each acronym 10 times. Person IV is a proficient signer who spelled at a speed similar to person I and II. In this experiment, we classified every acronym recorded by person IV with training data *T* containing transitions recorded by person I, II, and III using the method dependent on the acronym dictionary (similarly to the experiments presented in Table 6). The recognition rate was 81.8%. We performed similar experiments excluding particular signers from the training set. We then observed that person II had the highest (positive) impact on the results. Most likely this is caused by the fact that both person II and IV are proficient signers (unlike person I and III). This leads to the conclusion that gestures performed by proficient signers are better recognized with other proficient signers’ training data, and the same can be assumed in the case of inexperienced signers.

Recognition time of a single acronym depends strongly on its length (number of letters) and speed of fingerspelling. Hand segmentation and feature extraction of a single point cloud took approximately 55 ms on a PC with Intel Core i7-4710HQ, 2.5 GHz CPU, and 16 GB RAM, 800 MHz. Classification time of a single sequence was 10–13 ms. It depended on the number of states in HMM models (larger models require more calculations).

### 3.4. Recognition of Isolated Letters

The previous experiments were focussed on the fingerspelling considered as quick highly coarticulated motions. However, our hand shape representation method can also be applied for recognition of hand postures in single frames, but in this case, the classifier has to be replaced. In this subsection, we present experimental results of our method with SVM (support vector machine) [33] as a classifier of single hand postures. Since SVM can distinguish only between two classes, we used the all-versus-all method for multiclass classification. As a kernel, we used the function:

(12) $$H\left(x,x^{\prime }\right)=\exp(-\gamma \cdot |x-x^{\prime }{|}^{2}),$$

where $\gamma =1/FV_{size}$ and $FV_{size}$ is the size of the feature vector (48 in our case). We used the best combination of features FS2+(*E*,*C*,*L*,*S*) obtained in the experiments from Section 3.2. The classification was performed using Matlab software with LIBSVM (library for support vector machines) [34].

For these experiments, we used a publicly available American finger alphabet dataset [23]. It is a very challenging dataset consisting of 24 hand postures performed a variable number of times by 5 people, which amounts to 64,749 executions in total. For the classification, we used 400 depth maps for each gesture performed by each person, starting at file number 100. The offset from the beginning has been used due to accidentally captured head images (without a visible hand) or incorrect hand postures present in the first files in some cases. The hand postures represent the letters of the American finger alphabet without J and Z (since they involve motion). The gestures were recorded by a Kinect camera.

The results, presented in Table 7, were obtained using leave-one-person-out 5-fold cross-validation tests. Our approach is better than or comparable to other considered methods.

## 4. Conclusions

In the paper, we presented a method for recognition of static hand posture sequences in a form of fingerspelled letters of the Polish finger alphabet. The method works with 3D data in a form of point clouds. As classification features, we used point cloud descriptors: VFH, eigenvalues-based descriptors, and modified versions of ESF and GRSD. The hand shape representation based on such an approach can be applied both for the recognition of hand postures in single frames, which we confirmed on a challenging American finger alphabet dataset, and for the recognition of fingerspelling considered as quick highly coarticulated motions. In the second case, our method is based on a hidden Markov models classifier with the models trained by transitions between postures corresponding to two consecutive letters. We considered three transition models (B1, B2, B3) and two model networks: Dependent on and independent from the dictionary of acronyms.

We used our own dataset, consisting of training data in the form of transitions between letters and testing data in the form of acronyms. Using our own, publicly accessible dataset, we achieved high recognition rates for the method dependent on the acronym dictionary. The results obtained for the method independent from the acronym dictionary are promising; however, the problem of letter sequence classification without a dictionary still remains challenging and can be a subject for future work. Another future study topic may be expanding the recognition method by adding the possibility to recognize not only static but also dynamic letters of the finger alphabet.

## Figures and Tables

**Table 1:** Content of the prepared dataset (depth images registered by Kinect 2.0).

Name	Description
*T* (transitions)	$T=T_{I}\cup T_{II}\cup T_{III}(T_{p},p\in \left\{I,II,III\right\}$ performed by person *p*) $T_{p}$: 240 transitions, 5 executions Total: 240×5×3=3600 executions
*A* (acronyms)	$A=A_{I}\cup A_{II}\cup A_{III}(A_{p},p\in \left\{I,II,III\right\}$ performed by person *p*) $A_{p}$: 20 acronyms, 10 executions Total: 20×10×3=600 executions

**Table 2:** Recognition rates (sorted in descending order by mean rate) of the method independent from the acronym dictionary using single eigenvalues-based descriptors. The results were obtained for the model B2 with *T* as the training set and *A* as the testing set.

Eigenvalues-Based Descriptors	Recognition Rate [%]			
	Person I	Person II	Person III	Mean
E	50.8	31.5	58.4	46.9
O	47.5	18.7	50	38.7
C	38.8	25.7	50.6	38.4
L	38.4	27.8	41.4	35.9
S	33.4	22.5	48.4	34.9
A	33.4	22.5	48.4	34.9
P	30.4	26.2	28.2	28.3

**Table 3:** Recognition rates of the method independent from the acronym dictionary using different combinations of features. The results were obtained for the model B2 with *T* as the training set and *A* as the testing set.

Feature Set	Recognition Rate [%]			
	Person I	Person II	Person III	Mean
FS1	68.3	38.6	69.6	58.8
FS2	89.9	60.4	79.1	76.5
FS2 + *E*	90.3	65.9	79.5	78.6
FS2 + (*E*,*O*)	87.8	66	79.6	77.8
FS2 + (*E*,*O*,*C*)	87.8	66.4	80.6	78.3
FS2 + (*E*,*O*,*C*,*L*)	87.5	67.2	79.5	78.1
FS2 + (*E*,*O*,*C*,*L*,*S*)	88.1	67.1	80.2	78.5
FS2 + (*E*,*O*,*C*,*L*,*S*,*A*)	87.3	66.6	80.3	78.1
FS2 + (*E*,*O*,*C*,*L*,*S*,*A*,*P*)	86.3	66.7	80.2	77.7
FS2 + (*E*,*C*,*L*,*S*)	88.7	67.4	81.3	79.1

**Table 4:** Recognition rates of the method independent from the acronym dictionary using the best combination of features FS2 + (*E*,*C*,*L*,*S*) with *T* as the training set and *A* as the testing set.

Person	Recognition Rate [%]		
	B1	B2	B3
I	84.8	88.7	64.6
II	69	67.4	61.2
III	71.7	81.3	48.3
Mean	75.2	79.1	58
Standard deviation	6.9	8.8	7

**Table 5:** Recognition rates of the method using the acronym dictionary. Feature vector based on FS2 + (*E*,*C*,*L*,*S*). Training set: *T*, testing set: *A*.

Person	Recognition Rate [%]		
	B1	B2	B3
I	97.5	99.6	99.6
II	92.3	87.3	90.5
III	100	100	100
Mean	96.6	95.6	96.7
Standard deviation	3.2	5.9	4.4

**Table 6:** Recognition rates of the method using the acronym dictionary. The results were obtained by leave-one-person-out tests, i.e., with $T\backslash T_{p}$ as the training set and $A_{p}$ as the testing set for person *p*.

Person	Recognition Rate [%]		
	B1	B2	B3
I	85	88.2	76.8
II	83.6	80.7	77.5
III	97.3	96.4	92.3
Mean	88.6	88.4	82.2
Standard deviation	6.2	6.4	7.1

**Table 7:** Comparison between recognition rates of our approach using FS2 + (*E*,*C*,*L*,*S*) and methods found in the literature. The experiments were performed using leave-one-person-out 5-fold cross-validation tests.

Method	Recognition Rate [%]
Keskin et al. [35]	43
Pugeault et al. [36]	49
Pedersoli et al. [37]	56
Kuznetsova et al. [38]	57
Wang et al. [39]	58.3
Dalal et al. [1,40]	65.4
Dong et al. [41]	70
Zhang et al. [42]	73.3
Wang et al. [3]	75.8
Feng et al. [1]	78.7
our method	78.8

## References

[1] Feng B., He F., Wang X. (2016). Depth-Projection-Map-Based Bag of Contour Fragments for Robust Hand Gesture Recognition. IEEE Trans. Hum.-Mach. Syst..

[2] Li W.J., Hsieh C.Y., Lin L.F., Chu W.C. Hand gesture recognition for post-stroke rehabilitation using leap motion. Proceedings of the 2017 International Conference on Applied System Innovation (ICASI).

[3] Wang C., Liu Z., Chan S.C. (2015). Superpixel-Based Hand Gesture Recognition With Kinect Depth Camera. IEEE Trans. Multimed..

[4] Yang C. (2017). Static Gesture Recognition Algorithm Based on Upper Triangular Image Texture and Recursive Graph. J. Eng. Technol. Sci..

[5] Otiniano-Rodríguez K., Cámara-Chávez G. Finger Spelling Recognition from RGB-D Information Using Kernel Descriptor. Proceedings of the 2013 XXVI Conference on Graphics, Patterns and Images.

[6] Escobedo E., Camara G. Finger Spelling Recognition from Depth Data Using Direction Cosines and Histogram of Cumulative Magnitudes. Proceedings of the 2015 28th SIBGRAPI Conference on Graphics, Patterns and Images.

[7] Hu Y. (2018). Finger spelling recognition using depth information and support vector machine. Multimed. Tools Appl..

[8] Lamar M.V., Bhuiyan M.S., Iwata A. Hand alphabet recognition using morphological PCA and neural networks. Proceedings of the IJCNN’99. International Joint Conference on Neural Networks. Proceedings (Cat. No.99CH36339).

[9] Oz C., Leu M.C. (2005). Recognition of Finger Spelling of American Sign Language with Artificial Neural Network Using Position/Orientation Sensors and Data Glove. Proceedings of the Second International Conference on Advances in Neural Networks—Volume Part II.

[10] Goh P., Holden E.J. Dynamic Fingerspelling Recognition using Geometric and Motion Features. Proceedings of the 2006 International Conference on Image Processing.

[11] Kim T., Livescu K., Shakhnarovich G. American sign language fingerspelling recognition with phonological feature-based tandem models. Proceedings of the 2012 IEEE Spoken Language Technology Workshop (SLT).

[12] Kim T., Keane J., Wang W., Tang H., Riggle J., Shakhnarovich G., Brentari D., Livescu K. (2017). Lexicon-free fingerspelling recognition from video: Data, models, and signer adaptation. Comput. Speech Lang..

[13] Liwicki S., Everingham M. Automatic recognition of fingerspelled words in British Sign Language. Proceedings of the 2009 IEEE Computer Society Conference on Computer Vision and Pattern Recognition Workshops.

[14] Ricco S., Tomasi C. (2010). Fingerspelling Recognition through Classification of Letter-to-Letter Transitions. Computer Vision—ACCV 2009: 9th Asian Conference on Computer Vision, Revised Selected Papers, Part III.

[15] Wang T. (2015). Hidden Markov Model Based Recognition of German Finger Spelling Using the Leap Motion. Master’s Thesis.

[16] Kapuściński T., Oszust M., Wysocki M., Warchoł D. (2015). Recognition of Hand Gestures Observed by Depth Cameras. Int. J. Adv. Robot. Syst..

[17] Warchoł D., Wysocki M. (2016). Recognition of Hand Posture Based on a Point Cloud Descriptor and a Feature of Extended Fingers. J. Autom. Mob. Robot. Intell. Syst..

[18] Warchoł D. (2018). Hand posture recognition using modified Ensemble of Shape Functions and Global Radius-based Surface Descriptor. Comput. Sci..

[19] Rusu R.B., Bradski G.R., Thibaux R., Hsu J.M. Fast 3D recognition and pose using the Viewpoint Feature Histogram. Proceedings of the 2010 IEEE/RSJ International Conference on Intelligent Robots and Systems.

[20] Weinmann M., Jutzi B., Hinz S., Mallet C. (2015). Semantic point cloud interpretation based on optimal neighborhoods, relevant features and efficient classifiers. ISPRS J. Photogr. Remote Sens..

[21] Wohlkinger W., Vincze M. Ensemble of shape functions for 3D object classification. Proceedings of the 2011 IEEE International Conference on Robotics and Biomimetics.

[22] Marton Z.C., Pangercic D., Blodow N., Beetz M. (2011). Combined 2D-3D Categorization and Classification for Multimodal Perception Systems. Int. J. Robot. Res..

[23] Pugeault N. ASL Finger Spelling Dataset. http://empslocal.ex.ac.uk/people/staff/np331/index.php?section=FingerSpellingDataset.

[24] Bradski G., Kaehler A. (2008). Learning OpenCV: Computer Vision with the OpenCV Library.

[25] Rusu R.B., Cousins S. (2011). 3D Is Here: Point Cloud Library (PCL). Proceedings of the IEEE International Conference on Robotics and Automation (ICRA).

[26] Documentation for Hidden Markov Toolkit (HTK) Library. http://htk.eng.cam.ac.uk.

[27] Bouguet J.Y. (2015). Camera Calibration Toolbox for Matlab. http://www.vision.caltech.edu/bouguetj/calibdoc.

[28] Marton Z.C., Pangercic D., Rusu R.B., Holzbach A., Beetz M. Hierarchical Object Geometric Categorization and Appearance Classification for Mobile Manipulation. Proceedings of the 2010 10th IEEE-RAS International Conference on Humanoid Robots.

[29] Kraiss K.F. (2006). Advanced Man-Machine Interaction: Fundamentals and Implementation (Signals and Communication Technology).

[30] Rabiner L.R. (1990). Readings in Speech Recognition.

[31] The Kinect for Windows Software Development Kit. https://www.microsoft.com/en-us/download/details.aspx?id=44561.

[32] Theodoridis S., Koutroumbas K. (2008). Pattern Recognition.

[33] Vapnik V. (1995). The Nature of Statistical Learning Theory.

[34] Chang C.C., Lin C.J. (2011). LIBSVM: A library for support vector machines. ACM Trans. Intell. Syst. Technol..

[35] Keskin C., Kiraç F., Kara Y.E., Akarun L. Real time hand pose estimation using depth sensors. Proceedings of the 2011 IEEE International Conference on Computer Vision Workshops (ICCV Workshops).

[36] Pugeault N., Bowden R. Spelling it out: Real-time ASL fingerspelling recognition. Proceedings of the 2011 IEEE International Conference on Computer Vision Workshops (ICCV Workshops).

[37] Pedersoli F., Benini S., Adami N., Leonardi R. (2014). XKin: An Open Source Framework for Hand Pose and Gesture Recognition Using Kinect. Vis. Comput..

[38] Kuznetsova A., Leal-Taixé L., Rosenhahn B. Real-time sign language recognition using a consumer depth camera. Proceedings of the 2013 IEEE International Conference on Computer Vision Workshops, 3rd Workshop on Consumer Depth Cameras for Computer Vision (CDC4CV).

[39] Wang Y., Yang R. Real-time hand posture recognition based on hand dominant line using kinect. Proceedings of the 2013 IEEE International Conference on Multimedia and Expo Workshops (ICMEW).

[40] Dalal N., Triggs B. (2005). Histograms of Oriented Gradients for Human Detection. 2005 IEEE Computer Society Conference on Computer Vision and Pattern Recognition.

[41] Dong C., Leu M.C., Yin Z. American Sign Language Alphabet Recognition Using Microsoft Kinect. Proceedings of the 2015 IEEE Conference on Computer Vision and Pattern Recognition Workshops (CVPRW).

[42] Zhang C., Yang X., Tian Y. Histogram of 3D facets: A characteristic descriptor for hand gesture recognition. Proceedings of the 2013 10th IEEE International Conference and Workshops on Automatic Face and Gesture Recognition (FG).

